# Prevalence of Active Trachoma and Its Associated Factors among Rural and Urban Children in Dera Woreda, Northwest Ethiopia: A Comparative Cross-Sectional Study

**DOI:** 10.1155/2015/570898

**Published:** 2015-03-25

**Authors:** Metadel Alemayehu, Digsu N. Koye, Amare Tariku, Kedir Yimam

**Affiliations:** ^1^West Gojjam Zone Health Bureau, Finote Selam, Ethiopia; ^2^Department of Epidemiology and Biostatistics, Institute of Public Health, College of Medicine and Health Science, University of Gondar, P.O. Box 196, Gondar, Ethiopia; ^3^Department of Human Nutrition, Institute of Public Health, College of Medicine and Health Science, University of Gondar, P.O. Box 196, Gondar, Ethiopia; ^4^Department of Public Health, College of Medicine and Health Science, Debre Markos University, P.O. Box 269, Debre Markos, Ethiopia

## Abstract

*Background*. Trachoma is the most common infectious cause of blindness worldwide. Once an epidemic in most parts of the world, it has largely now disappeared from developed countries. However, it continues to be endemic in many developing countries like Ethiopia. Even if several studies were conducted in different parts of Ethiopia, most of them did not show the independent predictors for rural and urban children separately. Therefore, this study aimed at assessing the prevalence and associated factors of active trachoma in urban and rural children. *Methods*. Community based comparative cross-sectional study was conducted in Dera woreda. Multistage sampling technique was used to select 671 children of one up to nine years of age. Data were collected by face to face interview and observation using a structured and pretested questionnaire. Binary Logistic Regression Model was fitted to consider adding independent predictors of outcome. *Results*. Out of 671 children, 20 (9.3%) of urban and 85 (18.6%) of rural children were positive for active trachoma. Having discharge on eye (AOR = 6.9, 95% CI: 1.79–27.89), presence of liquid waste around the main house (AOR = 5.6, 95% CI: 1.94–16.18), and living in households without latrine (AOR = 4.39, 95% CI: 1.39–13.89) were significantly associated with active trachoma of urban children. Rural children who had discharge on their eye (AOR = 5.86, 95% CI: 2.78–12.33), those who had unclean face (AOR = 4.68, 95% CI: 2.24–9.81), and those living in households with feces around their main houses (AOR = 1.94, 95% CI: 1.04–3.62) were significantly associated with active trachoma. *Conclusion*. The result showed that the prevalence of active trachoma in urban areas of the district was below WHO threshold of 10% to determine trachoma as public health problem. However, in rural areas of the district it is far from elimination of trachoma as a public health problem. Thus, in order to improve awareness of the community there is a need of health education programs regarding facial cleanliness, utilization of latrine, and proper solid waste and liquid waste disposal using multidisciplinary approach.

## 1. Introduction

Trachoma, caused by* Chlamydia trachomatis*, is the most common infectious cause of blindness worldwide. Infection with trachoma is most commonly found in children and with repeated reinfection it can lead to scarring complications and blindness in late childhood and adult life. It is transmitted through the discharge from infected children eyes and passed on by hands (fingers), on clothes, or by flies that land on the eyes of noninfected child. It occurs in areas with poor personal and community hygiene (i.e., hot, dry, and dusty climate) and typically affects the most marginalized, deprived members of a community [1–3].

According to the World Health Organization (WHO), trachoma is currently responsible for more than 3% of the world's blindness [4]. Once an epidemic in most parts of the world, it has largely now disappeared from developed countries like Europe and North America. However, it continues to be endemic in many under developed countries. In Asia, some parts of Latin America, Africa, the Middle East, and Western Pacific, it is estimated that 320 million people live in endemic areas and 8 million people suffer from trachomatous trichiasis (TT) [2].

In Africa, 27.8 million cases of active trachoma (68.5% of all) and 3.8 million cases of trichiasis (46.6% of all) are found and it is believed to be endemic in 33 of the 56 countries in Africa. The highest prevalence of active trachoma and trichiasis remains in the Sahel area of West Africa and Savannah areas of East and Central Africa [5]. A high proportion of TF prevalence in 1–9-year-olds in South Sudan (83%), Ethiopia (64%), Guinea (50%), Uganda (37%), Chad (38%), Central Africa Republic (38%), and Tanzania (32%) [5, 6]. Studies in Gambia, Cameroon, and Nigeria also showed that the overall prevalence of active trachoma in children aged 1–9 years of age were 3.8%, 12%, and 37.7%, respectively [7–9].

Ethiopia is one of the five countries in which 49% of global burden of active trachoma is located. One nationwide survey on blindness, low vision, and trachoma reported that the national prevalence of active trachoma (either TF or TI) among children with age group of 1–9 years is 40.14% [10]. More recent studies conducted in Dangila town of Amhara region and Kersa district of Oromia region showed the prevalence of active trachoma among children age 1–9 years were 12% and 25.2%, respectively [11–13].

In view of this, Ethiopia launched the VISION 2020 Initiative in September 2002 and developed its own 20 years strategic plan to eliminate trachoma [11]. Several studies were conducted in different parts of Ethiopia. However, most of the studies are inconclusive about the independent predictors of active trachoma for rural and urban communities. Moreover, based on the nation-wide survey conducted in 2006 Dera woreda has the highest prevalence of active trachoma. Therefore, this study aimed at assessing difference in prevalence and associated factors of active trachoma in urban and rural children of Dera woreda, Northwest Ethiopia.

## 2. Methods

### 2.1. Study Design and Study Setting

Community based comparative cross-sectional study was conducted in Dera woreda from March to April 2014. Dera woreda is one of the 12 woredas in South Gondar Zone which is located in the Northwest part of Ethiopia. The current population size of the woreda is estimated to be 279, 847; of these 257, 343 of them were rural in residence. For administrative purpose the woreda is divided into 32 administrative units (Kebeles) in which three of them are urban.

### 2.2. Source and Study Population

All children in Dera woreda from one to nine years of age were the source population of this study. Children who are living in households selected by systematic sampling technique were included in this study. Children who were unable to undergo physical examination due to serious medical illness were excluded from the study.

### 2.3. Sample Size Determination

Sample size was determinedby using double population proportion formula, at 95% confidence interval, 80% power of the study, and 1 to 2 ratio. The proportions were taken at 25.2% rural and 12% urban prevalence of active trachoma among children from one up to nine years of age which was conducted in Kersa district and Dangila town, respectively [12, 13]. The sample size was calculated by STATCALC program of EPI INFO version 1.1.7.14. By considering design effect of two and nonresponse rate of 10%; the final sample size of the study was 231 and 464 children for urban and rural kebeles, respectively.

### 2.4. Sampling Procedure

Multistage stratified sampling technique was used to select the study participants. At first stage, six out of 29 rural and two out of three urban kebeles were used based on the household size of the kebele and the sample kebeles were selected by lottery method. At second stage, households from each kebele were selected by systematic random sampling technique and the sample size in each kebele was determined by proportional allocation based on the. Lottery method was used to select a child in houses which had more than one child with the age of 1–9 years.

### 2.5. Variables of the Study

*Dependent variable* is the prevalence of active trachoma.* Independent variables* are sociodemographic characteristics (sex, age and marital status of the family head, wealth, religion, educational status of father and mother, occupation, and being model household of the health extension package), child characteristics (sex and age of child, frequency of washing face, using soap for washing, discharge on the eye, facial cleanness, number of fly in child face, and number of Zithromax prophylaxes dose used), and environmental factors (water source, latrine, waste disposal sites, etc.).

### 2.6. Operational Definition

*Model household*: households have successfully completed the 16 HEP packages and learn 96 recommended hour by health extension worker.* Active trachoma*: TF has been suggested by WHO as the key indicator for assessing the public health importance of active trachoma [14]. Hence, it was defined as the presence of at least five or more follicles in the upper tarsal conjunctiva each at least 0.5 mm in size.

### 2.7. Data Collection

Data were collected by face to face interview and observation using a structured and pretested questionnaire. The questionnaire was first prepared in English and translated to Amharic, and then it was again translated back to English by another person. A total of 15 health professionals were selected as data collector and four supervisors were assigned to the data collection for house hold head interviews. Trachoma grading was examined by 5 eye integrated worker (4 health officers and 1 diploma nurse), by wearing 2.5x loupes, assessed each eye for signs of active trachoma using the WHO simplified grading scheme. One supervisor (optometry) for trachoma grade examination supervision from University of Gondar was used.

The quality of data was assured by proper designing and pretesting of the questionnaires in one of the Kebeles other than the selected kebeles with similar sociodemographic characteristics. Training was given for both data collectors and supervisors by the principal investigator for three days before the pretest. Findings of the pretests were discussed during the training day and all the concerns were clarified. Every day after data collection, questionnaires were reviewed and checked for completeness by the supervisors and principal investigator and the necessary feedback was offered to the data collectors in the next morning.

### 2.8. Data Processing and Analysis

The data were cleaned to check for its completeness, consistency, and the presence of missed values. Then, it was entered into Epi Info version 7.1.1.4 and exported to SPSS version 20 for analysis. Descriptions of the main findings were done using frequencies, percentages, and summary statistics. Binary Logistic Regression Model was fitted to assess factors associated with active trachoma. Variables with *P* value < 0.2 in bivariate analysis were included in multivariate analysis. Those variables with *P* value < 0.05 in the multivariate analysis were considered as independent predictors for active trachoma. Odds ratio and 95% confidence interval were also reported.

### 2.9. Ethical Consideration

Ethical clearance was obtained from Institutional Review Board of University of Gondar. Permissions letter was also taken from Amhara Regional Research and Technology Transfer Office, South Gondar Zonal Health Department, Dera Woreda Health Office, and selected kebeles. Child assent and informed verbal consent were obtained from the sampled children and their parents. The respondents were also informed that they have full right to withdraw or refuse at any time from the process. Confidentiality of information given by each respondent was kept properly and anonymity was explained clearly for participant. Tetracycline eye ointment was provided to those who were diagnosed with active trachoma. Patients with trichiasis were referred to the nearby health center for further investigation and treatment.

## 3. Results

### 3.1. Characteristics of Respondents

A total of 215 urban and 456 rural children were participated in this study, which makes the response rate 96.5%. Out of these, 613 (91.4%) of them were from a married family. Regarding the educational status of their parents, 487 (73.5%) of fathers and 530 (79%) of mothers who live in the urban cannot read and write as compared to 370 (76%) fathers and (75.3%) mothers who live in the rural area. The majority, 638 (95.1%) of respondents, were orthodox in religion. More than two-third of child families 477 (71.1%) did graduate as model house hold for health extension package, 127 (59.1%) from urban and 350 (76.8%) from rural households (Table 1).

### 3.2. Characteristics of Children

Of 671 children, 351 (52.3%) of them were males. Out of the 671 children, 34 (15.8%) of urban and 241 (35.9%) of rural children were washing their face only once per day. One hundred forty-nine (69.3%) of urban and 130 (28.5%) of rural children were washing their faces by using soap. Of 671 children, about 14 (6.5%) of urban and 90 (20%) of rural children had discharge on their face. Regarding cleanliness, 121 (26.5%) of rural and 21 (9.8) of urban children had clean face. Majority of, 210 (97.6%) urban and 450 (98.6%) rural, children received at least one dose of Zithromax prophylaxis (Table 2).

### 3.3. Environmental and Housing Condition of the Households

Among rural households, 202 (44.3%) were getting drinking water from protected well, 61 (15.4%) were traveling more than 30 minutes to fetch water for their daily consumption and 93 (20.4%) were not getting water all year round. Of urban households, 189 (87.9%) were getting drinking water from pipe line and nearly all (99.5%) were traveling less than 30 minutes to fetch water for their daily consumption and 45 (20.9%) were not getting water all year round (Table 3).

The average daily water consumption of families was found to be 9.43 liters/person/day and 8.34 liters/person/day for urban and rural households, respectively. Among selected households, 87 (40.5%) of urban and 143 (31.4%) of rural households have solid waste disposal pit. Of these, 83 (95.4%) of urban and 117 (81.8%) of rural solid waste disposal pits were functional. In the study area, 70 (32.6%) of urban and 68 (14.9%) of rural households have liquid waste disposal pits. At the time of the study, 428 (63.8%) households had functional latrine. Of these, 187 (98.9%) were from urban and 241 (86.1%) were from rural households. The available latrines in the child families were utilized by both adults and children in 131 (70.1%) of urban and 165 (68.5%) of rural households.

### 3.4. Prevalence of Active Trachoma

**Figure 1:**
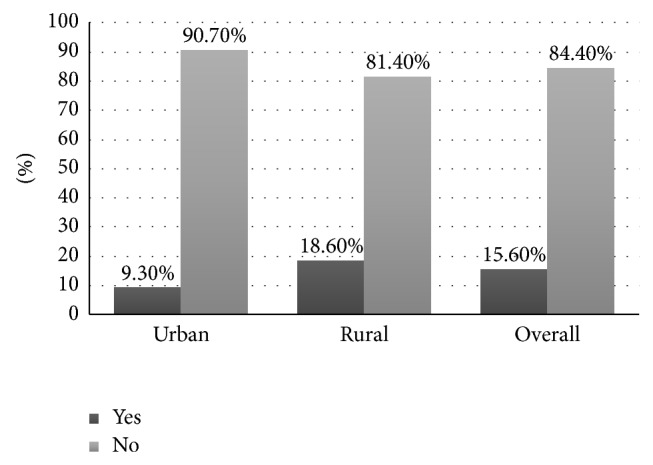
Prevalence of active trachoma among children aged one to nine years in Dera woreda, Northwest Ethiopia, 2014.

Out of 671 children who were screened for active trachoma, 105 (15.6%) of them with 95% CI [12.8–18.3] had active trachoma. Of these, 20 (9.3%) and 85 (18.6%) were from urban and rural children, respectively (Figure 1).

### 3.5. Factors Associated with Active Trachoma in Urban Children

Children who had discharge on their eye were 6.9 times more likely to have active trachoma as compared to those who had no discharge on their eye (AOR = 6.9, 95% CI: 1.79–27.89). The likelihood of active trachoma among children who were from households with liquid waste around their main house was higher (AOR = 5.6, 95% CI: 1.94–16.18) as compared to their counterparts. The odds of active trachoma among children who were from households without latrine were higher (AOR = 4.39, 95% CI: 1.39–13.89) as compared to their counterparts (Table 6).

### 3.6. Factors Associated with Active Trachoma in Rural Children

Rural children who had discharge on their eye were 5.8 times more likely to have active trachoma as compared to those who had no discharge on their eye (AOR = 5.86, 95% CI: 2.78–12.33). Those rural children who have unclean face had higher (AOR = 4.68, 95% CI: 2.24–9.81) odds of active trachoma than their counter parts. Children who were living in the households which had feces near their main house were 1.94 times (AOR = 1.94, 95% CI: 1.04–3.62) more likely to have active trachoma as compared to their counter parts.

### 3.7. Factors Associated with Active Trachoma in Dera Woreda

In order to identify independent predictors for the overall prevalence statistical model was fitted. Children with discharge on their eye were 5.31 times more likely to have active trachoma than those without discharge (AOR = 5.3195% CI: 2.71–10.4). The likelihood of active trachoma among children with unclean faces was higher (AOR = 4.04 95% CI: 2.11–7.73) as compared to those with clean faces. In addition, children who live in vicinity which have feces around their main houses were 3 times more likely to have active trachoma than children who live in vicinity which have not feces around their main house (AOR = 2.7; 95% CI: 1.53–4.78).

## 4. Discussion

Although trachoma is avoidable, it remains a neglected public health issue owing to few voices speaking out on behalf of people affected by trachoma [15]. Despite the implementation of SAFE strategy (surgery for trichiasis, antibiotics for active disease, facial hygiene, and environmental improvement) to reduce the transmission of the disease in Ethiopia, the prevalence of trachoma is still one of the highest in the region [10]. Therefore, this study helps to identify factors associated with active trachoma for urban and rural children separately.

The study revealed that the prevalence of active trachoma in the Dera woreda was found to be 18.6% among rural and 9.3% among urban children. The prevalence of active trachoma among rural children was found to be much higher than urban children. This could be explained by the poor access of health services and presence of poor hygiene in rural households. For instance, in this study, the availability and utilization of latrine in urban households was much higher (87%) than the rural households (52.8%). It could also be explained by absence of separate home for cattle's, utilization of cattle dung for energy and presence of garbage and feces around the home in rural households.

The overall prevalence of active trachoma was low as compared to other studies conducted in Ankober, North Shewa (53.9%), Baso Liben, West Gojjam (24.1%), and Kersa, Jimma Zone (25.2%) of Ethiopia. This discrepancy in the magnitude of active trachoma may be due to latrine availability (24% in Ankober, 10% in Kersa) and poor availability and accessibility of water (89% of study households in Kersa travel >30 minutes walking distance to get water) [13, 16, 17]. The finding of the presence study was much higher than other African studies conducted in Gambia (3.8%), Sierra Lion (5%), Cameroon (11.2%), and Malawi (13.6%). The difference could be explained by the following reasons: the study conducted in Sierra Lion reported low prevalence of unclean face and good personal hygiene. In Gambia there was 98% latrine access, 97% of them had disposal pit, and 93% of households move less than 30 minutes to get water. In Cameroon there was high coverage of mass drug administration [7, 8, 18, 19]. However, this finding was found to be lower as compared to African studies conducted in Nigeria (37.7%) and Niger (23.4%) [9, 20]. This could be due to mass drug distribution and difference in personal and environmental factors including latrine availability.

The result of this study showed that rural children with unclean faces were 4.7 times more likely to have active trachoma than children with clean faces. This was in line with other similar studies conducted in Ethiopia [11, 17, 21] and Nigeria [9]. This could be explained by poor access of water supply in rural areas; average water consumption of the study area was 8.6 liters per day per person, which is much lesser than the WHO standard 20 liters per day per person.

In both urban and rural children, having discharge on eye was significantly associated with active trachoma. Children who had discharge on their eye were more likely to have active trachoma as compared to those who had no discharge. Center for International Health in 2007 identifies six Ds to make memorization of risk factors of trachoma easier. These are dry, dusty, dirty, dung, discharge, and density (overcrowding in the home) which could be an explanation for the above finding [22].

In this study, availability of latrine among urban households was significantly associated with the prevalence of active trachoma among urban children. Urban children who were living in households without latrine were 4.4 times more likely to have active trachoma as compared to their counterparts. This finding was in congruent with other similar local and global studies [12, 23–25]. This could be explained by the presence of latrine at the household level that may reduce eye-seeking flies in the surrounding environment.

The presence of liquid waste around the main house had significantly associated with prevalence of active trachoma among urban children (Table 4). Children from urban households with liquid waste around the main house were 5.6 times more likely to have active trachoma as compared to their counterparts. This could be explained by the likelihood of the presence of liquid waste around the main house was higher and persistent in urban area than rural area. The presence of small scale industrial ecology in the urban area makes the liquid wastes more persistent and long lasting which is favorable environmental condition for the breeding of trachoma vectors.

The presence of feces around the main house had significantly associated with prevalence of active trachoma among rural children. Children from rural households who had feces around the main house were nearly 2 times more likely to have active trachoma. This finding is in line with study done in China; children had a 2.5 times higher risk of active trachoma if they lived in households that reported defection close to the house [26]. The usual method of human feces disposal in rural households of the study area is open defecation in the bush between households and surrounding the villages. Human feces and, to a lesser extent, cattle dung are known to be the preferred breeding media for the fly vector of trachoma since isolated human feces on the soil surface are the best larval medium for Muscasorbens, the vector for trachoma, which could be an explanation for the above findings [27] (Table 5).

## 5. Conclusion

The overall prevalence of active trachoma in Dera woreda was lower than WHO threshold prevalence of 20% which is used to determine trachoma as a severe public health problem [26]. However, it is far from the elimination of trachoma as a public health problem in a community as when there is less than 5% clinical activity in children [27]. This study also revealed that the prevalence of active trachoma was found to be higher among rural children than urban children. Discharge on faces, cleanliness of child faces, and feces around the main house were independent predictors of active trachoma among rural children. Presence of discharge on faces, availability of latrine, and presence of liquid waste around the main house were significantly associated with active trachoma of urban children. Moreover, having unclean face, presence of discharge on faces, and feces around the main house were significantly associated with the overall prevalence of active trachoma in Dera woreda. Thus, there is a need of health education programs about facial cleanliness, use of latrine, and proper solid waste and liquid waste disposal using multidisciplinary approach.

## Figures and Tables

**Table 1:** Characteristics of respondent (child's father and mother) in Dera woreda, Northwest Ethiopia, 2014.

Variables	Urban	Rural	Total
Marital status			
Married	182 (84.6%)	431 (94.5%)	613 (91.4%)
Divorced	25 (11.6%)	18 (4.0%)	43 (6.4%)
Single	4 (1.9%)	0 (0%)	4 (0.6%)
Widowed	4 (1.9%)	7 (1.5%)	11 (1.6%)
Family size			
≤4	113 (52.6%)	219 (48%)	332 (49.5%)
>4	102 (47.4%)	237 (52%)	339 (50.5%)
Religion			
Orthodox	187 (87%)	451 (98.9%)	638 (95.1%)
Muslim	28 (13%)	5 (1.1%)	33 (4.9%)
Father educational status			
Cannot read and write	91 (42.7%)	396 (88%)	487 (73.5%)
Read/write	37 (17.4%)	50 (11.1%)	87 (13.1%)
Primary	30 (14.1%)	3 (0.7%)	33 (5%)
Secondary	21 (9.9%)	1 (0.2%)	22 (3.3%)
Preparatory and above	33 (15.6%)	1 (0%)	34 (5.1%)
Mother educational status			
Cannot read/write	103 (48.4%)	427 (93.8%)	530 (79.3%)
Read and write	38 (17.8%)	25 (5.5%)	63 (9.4%)
Primary school	38 (18.3%)	1 (0.2%)	40 (6%)
Secondary	10 (4.7%)	2 (0.4%)	12 (1.8%)
Preparatory and above	23 (10.8%)	0 (0%)	23 (3.5%)
Wealth index			
Poor	55 (25.6%)	109 (23.9%)	164 (24.4%)
Medium	82 (38.1%)	159 (34.9%)	241 (35.9%)
Rich	78 (36.3%)	188 (41.1%)	266 (39.7%)
Model household graduates			
Graduated	127 (59.1%)	350 (76.8%)	477 (71.1%)
Not graduated	88 (40.9%)	106 (23.2%)	194 (28.9%)

**Table 2:** Face washing and facial condition of children in Dera woreda, Northwest Ethiopia, 2014.

Variables	Urban	Rural	Total
Face wash per day			
Once	34 (15.8%)	261 (57.2%)	295 (44%)
Twice	92 (42.8%)	149 (32.7%)	241 (35.9%)
More than two times	89 (41.4%)	46 (10.1%)	135 (20.1%)
Use soap			
Yes	149 (69.3%)	130 (28.5%)	279 (41.6%)
No	66 (30.7%)	326 (71.5%)	392 (58.4%)
Face washing per day using soap			
Once	70 (47%)	92 (70.8%)	162 (58.1%)
Twice	36 (24.2%)	29 (22.3%)	65 (23.3%)
More than two times	43 (28.9%)	9 (6.9%)	52 (18.6%)
Discharge on the eye			
Yes	14 (6.5%)	91 (20%)	105 (15.6%)
No	201 (93.5%)	369 (80%)	566 (84.4%)
Condition of the child's face			
Clean	194 (90.2%)	335 (73.5%)	529 (78.9%)
Unclean	21 (9.8%)	121 (26.5%)	142 (21.1%)
Number of flies on the face			
None	104 (48.5%)	198 (43.5%)	302 (45%)
1–3	92 (42.7%)	187 (41%)	279 (41.5%)
4–7	17 (7.9%)	68 (14.8)	85 (12.7%)
>7	2 (0.9%)	3 (0.7%)	5 (0.8)
Zithromax prophylaxis			
None	6 (2.3%)	5 (1%)	11 (1.6%)
At least one dose	210 (97.7%)	450 (99.0%)	660 (98.4%)

**Table 3:** Environmental and housing condition of selected households in Dera woreda, Northwest Ethiopia, 2014.

Variables	Urban	Rural	Total
Travelling time to water source			
Less than 30 minutes	214 (99.5%)	395 (86.6%)	609 (90.8%)
More than 30 minutes	1 (0.5%)	61 (15.4%)	62 (9.2%)
Solid waste disposal pit			
Yes	87 (40.5%)	143 (31.4%)	230 (34.3%)
No	128 (59.5%)	313 (68.6%)	441 (65.7%)
Condition of solid waste disposal pit			
Functional	83 (95.4%)	117 (81.8%)	200 (87%)
Nonfunctional	4 (4.6%)	26 (18.2%)	30 (13%)
Garbage near the main house			
Yes	68 (31.6%)	196 (43%)	264 (39.3%)
No	147 (68.4%)	260 (57%)	407 (60.7%)
Liquid waste disposal pit			
Yes	70 (32.6%)	68 (14.9%)	138 (20.6%)
No	145 (67.4%)	388 (85.1%)	533 (79.4%)
Condition of liquid waste disposal pit			
Functional	69 (98.6%)	59 (86.8%)	128 (92.8%)
Nonfunctional	1 (1.4%)	9 (13.2%)	10 (7.2%)
Liquid wastes near the main house			
Yes	42 (19.5%)	137 (30%)	179 (26.7%)
No	173 (80.5%)	319 (70%)	492 (73.3%)
Latrine availability			
Yes	189 (87.9%)	280 (61.4%)	469 (69.9%)
No	26 (12.1%)	176 (38.6%)	202 (30.1%)
Latrine utilization			
Yes	187 (98.9%)	241 (86.1%)	428 (91.2%)
No	2 (1.1%)	39 (13.9%)	41 (8.74%)
Who uses the latrine in the families			
Only adults	56 (29.9%)	76 (31.5%)	132 (30.8%)
Adults and children	131 (70.1%)	165 (68.5%)	296 (69.2%)
Human faces around the main house			
Yes	22 (10.2%)	112 (24.6%)	134 (20%)
No	193 (89.8%)	344 (75.4%)	537 (80%)

**Table 4:** Factors associated with active trachoma among urban children in Dera woreda, Northwest Ethiopia 2014.

Variables	Active Trachoma		Crude OR (95% CI)	Adjusted OR (95% CI)
	Yes	No		
Discharge on the eye				
Yes	5 (25%)	9 (4.6%)	7.07 (2.05–23.18)	**6.98 (1.79–27.9)** ^***^
No	15 (75%)	186 (95.4%)	1	1
Liquid waste around the main house				
Yes	12 (60%)	30 (15.4%)	8.25 (3.11–21.89)	**5.60 (1.94–16.18)** ^**^
No	8 (40%)	165 (84.5%)	1	1
Availability of latrine				
Yes	12 (60%)	177 (90.8%)	1	1
No	8 (40%)	18 (9.2%)	6.56 (2.37–18.14)	**4.39 (1.39–13.89)** ^*^

**Table 5:** Factors associated with active trachoma among rural children in Dera woreda, Northwest Ethiopia 2014.

Variables	Active trachoma		Crude OR (95% CI)	Adjusted OR (95% CI)
	Yes	No		
Discharge on the eye				
Yes	55 (64.7%)	35 (9.4%)	17.6 (10.01–30.96)	**5.86 (2.78–12.33)** ^**^
No	30 (35.3%)	336 (90.6%)	1	
Condition of face				
Clean	23 (27.1%)	312 (84.1%)	1	
Unclean	62 (72.9%)	59 (15.9%)	14.28 (8.19–24.79)	**4.68 (2.24–9.81)** ^**^
Feces around the main house				
Yes	32 (37.6%)	80 (21.6%)	2.19 (1.33–3.64)	**1.94 (1.04–3.62)** ^**^
No	53 (62.4%)	291 (78.4%)	1	

**Table 6:** Factors associated with active trachoma among children in Dera Woreda, Northwest Ethiopia 2014.

Variables	Active trachoma		Crude OR (95% CI)	Adjusted OR (95% CI)
	Yes	No		
Condition of face				
Clean	38 (36.2%)	491 (86.7%)	1	1
Unclean	67 (63.8%)	75 (13.3%)	11.54 (7.24–18.40)	**4.04 (2.11–7.73)** ^**^
Discharge on the eye				
No	45 (42.9%)	522 (92.2%)	1	1
Yes	60 (57.1%)	44 (7.8%)	15.82 (9.65–25.92)	**5.31 (2.71–10.4)** ^**^
Feces around the main house				
Yes	43 (41%)	91 (16.1%)	3.62 (2.31–5.67)	**2.7 (1.53–4.78)** ^*^
No	62 (59%)	475 (83.9)	1	
